# Impact of Immune-Related Adverse Events on Immune Checkpoint Inhibitors Treated Cancer Patients’ Survival: Single Center Experience and Literature Review

**DOI:** 10.3390/cancers15030888

**Published:** 2023-01-31

**Authors:** Raquel Romão, Ana S. Mendes, Ridhi Ranchor, Maria João Ramos, João Coelho, Rita Carrilho Pichel, Sérgio Xavier Azevedo, Paula Fidalgo, António Araújo

**Affiliations:** 1 Medical Oncology Department, Centro Hospitalar Universitário do Porto, 4099-001 Porto, Portugal; 2 Oncology Research Unit, UMIB-Unit for Multidisciplinary Research in Biomedicine, ICBAS-School of Medicine and Biomedical Sciences, Universidade do Porto, 4050-346 Porto, Portugal

**Keywords:** immune checkpoint inhibitors, immune-related adverse event, survival, prognosis

## Abstract

**Simple Summary:**

The widespread use of immune checkpoint inhibitors (ICI) came along with a new challenge for oncologists, immune-related adverse events (irAE). A positive correlation between irAE onset and ICI efficacy has been suggested. However, it remains unsettled. Whether the association exists and if it is affected by cancer type or ethnicity needs further investigation. This study provides additional evidence to support this association by using a retrospective, single-center cohort design to analyze survival outcomes and the development of irAEs of 155 patients. Overall, the study offers new insights into the potential use of irAEs as biomarkers for response and survival in solid tumor patients receiving ICIs, and highlights the need for further research in this area.

**Abstract:**

Immune-related adverse events have emerged as a new challenge and its correlation with survival remains unclear. The goal of our study was to investigate the effect of irAE on survival outcomes in solid tumor patients receiving ICI treatment. This was a retrospective, single-center study at a university hospital involving patients with malignancy who received immune checkpoint inhibitors. Chart review was performed on each patient, noting any irAE, including new events or worsening of previous autoimmune condition after starting treatment with ICI. A total of 155 patients were included, 118 (76.1%) were male, with median age of 64 years. Median follow up time was 36 months. Seventy patients (45.2%) had at least one irAE. Of all irAE, nine (8.1%) were classified as grade 3 or higher according to the CTCAE version 5.0. There was one death secondary to pneumonitis. Median ICI cycles until first irAE onset was 4 (range: 2–99). The objective response rate was higher for patients who developed irAE (18.7% vs. 9.0%; *p* = 0.001), as was median overall survival (18 months (95% CI, 8.67–27.32) vs. 10 (95% CI, 3.48–16.52) months; *p* < 0.016) and progression free survival (10 months (95% CI, 5.44–14.56) vs. 3 months (95% CI, 1.94–4.05); *p* = 0.000). The risk of death in patients with irAE was 33% lower when compared to patients without such events (hazard ratio (HR): 0.67; 95% CI, 0.46–0.99; *p* = 0.043). Development of irAE predicted better outcomes, including OS in patients with advanced solid tumors treated with ICI. Further prospective studies are needed to explore and validate this prognostic value.

## 1. Introduction

Over the last decade, immunotherapy has transformed cancer treatment, improving the prognosis of multiple types of malignancies. Among the different forms of immunotherapy, immune checkpoint inhibitors (ICI) showed remarkable benefits and durable clinical responses in certain patients [1].

Immune checkpoint inhibitors are immunomodulatory antibodies that target inhibitory T cell receptors, enhancing anti-tumor immune response [2]. The most distinguished ICI include cytotoxic T lymphocyte-associated protein 4 (CTLA-4) inhibitor (e.g., ipilimumab), anti-programmed cell death 1 (PD-1) (e.g., nivolumab and pembrolizumab), and anti-programmed cell death-ligand 1 (PD-L1) agents (e.g., atezolizumab, durvalumab and avelumab) [3]. This field is rapidly evolving, with new agents targeting other inhibitory T-cell, such as Lymphocyte-activation gene 3 (LAG-3), T-cell immunoglobulin, and mucin domain-containing 3 (TIM-3), or T-cell immune receptor with immunoglobulin and ITIM domain (TIGIT), being developed to further improve the effectiveness of cancer immunotherapy. These new agents aim to overcome the mechanisms of T-cell exhaustion, which can occur in cancer patients [4].

With the increasing use of these drugs in clinical practice, a new challenge has emerged, immune-related adverse events (irAE) [5]. As this drug class works by increasing host immune response, destabilizing immune homeostasis, and enhancing proinflammatory activity, it can induce inflammatory drug-related side effects. However, the pathophysiology underlying these events is not fully understood [6].

In the context of irAE, any organ or system can be affected. The most frequently described organs are the skin, gut, endocrine glands, liver, and lungs.The type of organ affected, seems to be related with the type and mechanism of ICI. For instance, colitis seems most frequent with anti-CTLA-4 and pneumonitis with anti-P-D1/PD-L1 antibodies. These toxicities are often reported as self-limited and easily manageable, though in a small portion they can be lethal [7]. The management of irAEs is not entirely defined, but there are some guidelines defining strategies on the basis of specific organ toxicity, to improve patient care and prevent life-threatening events [8,9].

Although ICI has been successful in improving response rates, it has been shown that only a small percentage of patients, approximately 20%, have benefited from treatment with CTLA-4 or PD-1 inhibitors [10,11].

The interest in predictive response biomarkers to ICI has grown substantially. PD-L1 expression has been found to show a positive association with response to anti-PD-1/PD-L1 antibodies, but it has also been acknowledged as a flawed indicator. Other biomarkers that have been found to predict response to ICI include the presence of high neoantigen loads and microsatellite instability (MSI) associated with mutations in mismatch repair (MMR) proteins. Additionally, studies have shown that intestinal microbial composition also plays a role in the therapeutic effects of ICI. These biomarkers have proven to be of great importance in predicting the efficacy of ICI, but more studies are needed to determine their full reliability and applicability in the clinical setting. Although ongoing efforts are being made to discover new biomarkers that can enhance the prediction of response to ICI, clinical biomarkers have received less attention in research [12].

Based on the ICI mechanism of action, it has been suggested that irAE onset may exemplify a clinical biomarker for ICI response. There was a suggestion of some association between irAE incidence and better clinical outcomes with ICI, but so far it remains unclear. Meanwhile, some publications have verified a positive correlation across different types of cancer between irAE development and longer survival [13,14]. Other investigations in the area have demonstrated unfauvorable results [15]. If the association exists, whether it will be affected by cancer type, organ-specific irAE, or ethnicity also needs to be explored.

The objective of this study was to evaluate the incidence and potential of irAE as biomarkers for response and survival in solid tumor patients receiving ICI in a palliative setting.

## 2. Materials and Methods

### 2.1. Patient Population

This was a retrospective, single-center cohort study of patients aged 18 years or older, with histologically or cytologically confirmed metastatic solid tumors who received at least one dose of ICI (Pembrolizumab, Nivolumab, Atezolizumab and Ipilimumab) as a single therapy, administered intravenously, at Centro Hospitalar Universitário do Porto from July 2012 to January 2020.

Patients with prior ICI therapy and patients with incomplete data were excluded.

### 2.2. Data Collection

Demographic data, such as patient’s age, Eastern Cooperative Oncology Group (ECOG) performance status, diagnosis date, date of widespread disease confirmation, histology type, prior therapeutic regimens, number of doses of ICI, tumor response based on Response Evaluation Criteria in Solid Solid Tumors version 1.1, progression date, and death date or last follow-up visit, were all retrieved from the digital health records. Additionally, irAEs were registered and graded according to Common Terminology Classification Adverse Events (CTCAE) version 5.0, as well the date of the diagnosis and the respective prescribed treatment.

### 2.3. Statistical Analysis

Standard descriptive statistics were used to summarize all variables. The Fisher exact test was used to determine the association between categorical variables. Objective response rate (ORR) was defined as the percentage of patients who have a confirmed complete response (CR) or partial response (PR) to the treatment.

The overall survival (OS) was defined as the time period from the initial administration of ICI therapy until death. The progression-free survival (PFS) was defined as the time period from the initial administration of ICI therapy until the documentation of tumor progression or death, whichever occurred first. At the end of the follow-up period, patients who were still alive or had not shown progression of their tumor were considered censored in the analysis.

Survival curves were generated by using the Kaplan–Meier product limit method, and differences in OS where analyzed by stratifying for the ocorrence of irAE using the log-rank test. Univariate and multivariate Cox regression were used to identify factors with potential prognostic significance. 

IBM SPSS Statistics 27.00.00 was applied for statistical analysis, and *p* < 0.05 was considered significant.

## 3. Results

### 3.1. Patient and Tumor Characteristics

The study included a total of 155 Caucasian patients who received ICI therapy. The median age of the patients was 64, and out of the total number of patients, 118 (76.1%) were male. Only two patients in the cohort had a previous history of autoimmune diseases (vitiligo and psoriasis). The study population was composed of patients with lung cancer (n = 76, 49%), melanoma (n = 28, 18.1%), renal cancer (n = 18, 11.6%), head and neck cancer (n = 17, 11%), bladder cancer (n = 11, 7.1%), and other types of cancer (n = 5, 3.2%). A total of 146 (94.2%) patients received anti-PD-1/PD-L1 drugs and only 9 (5.8%) patients received anti-CTLA-4. Out of the total number of patients, 80 (51.6%) were treated with pembrolizumab, 60 (38.7%) with nivolumab, nine (5.8%) with ipilimumab, and six (3.9%) with atezolizumab. The median duration of treatment was six months (range, 0–63 months).

At the initiation of ICI therapy, 101 (65.2%) patients had a performance status of ECOG 1, 38 (24.5%) had a performance status of ECOG 0, and 16 (10.3%) had a performance status of ECOG 2. ICI therapy was used as first-line treatment in 50 (32.3%) patients, second-line in 91 (58.7%), third-line in 11 (7.1%), and fifth or later line in 3 (1.9%) patients. The median progression-free survival (PFS) was 5 months (95% CI, 3.2–6.8) and the median overall survival (OS) was 15 months (95% CI, 11.23–18.77). A total of 43 (27.7%) patients had an objective response and 71 (45.8%) patients had disease control (complete/partial response or stable disease). The objective response rate by cancer type was 36% for lung cancer, 14% for melanoma, 20% for renal cancer, 13% for head and neck cancer, 83% for bladder cancer, and 40% for other cancers.

Patients’ characteristics at baseline in the whole cohort and specified by development or not of irAE are summarized in Table 1.

### 3.2. Immune-Related Adverse Events

In this study, 70 (45.2%) patients developed irAE, with 25 (16.1%) experiencing more than one event. The median number of ICI cycles before an irAE onset was four (range, 2–99). When examining irAE by organ system, 34 (35.4%) patients had dermatological events, 17 (17.7%) had rheumatologic events, 14 (14.7%) had endocrine events, 13 (13.5%) had neurological or musculoskeletal events, nine (9.4%) had gastrointestinal and hepatic and biliary events, eight (8.3%) had pulmonary events, and one (1.0%) had a renal event (Table 2). 

The majority (92.9%) of irAE were grade 1 or 2. The only treatment-related death was caused by pneumonitis. 

Most irAE were managed with supportive care, but 27 (38.6%) cases required oral steroids and five (7.1%) required intravenous steroids. In a few cases, treatment escalation with methotrexate or immunoglobulin was used for patients with severe arthritis or necrotizing inflammatory myositis. 

ICI was suspended in all patients with irAE grade 3 or more. One patient with preexisting psoriasis experienced a flare of the disease, but ICI treatment was not suspended.

### 3.3. Response Rate and Survival Analysis 

It was found that the ORR was higher for patients who developed irAE (18.7% vs. 9.0%; *p* = 0.001).

**Figure 1:**
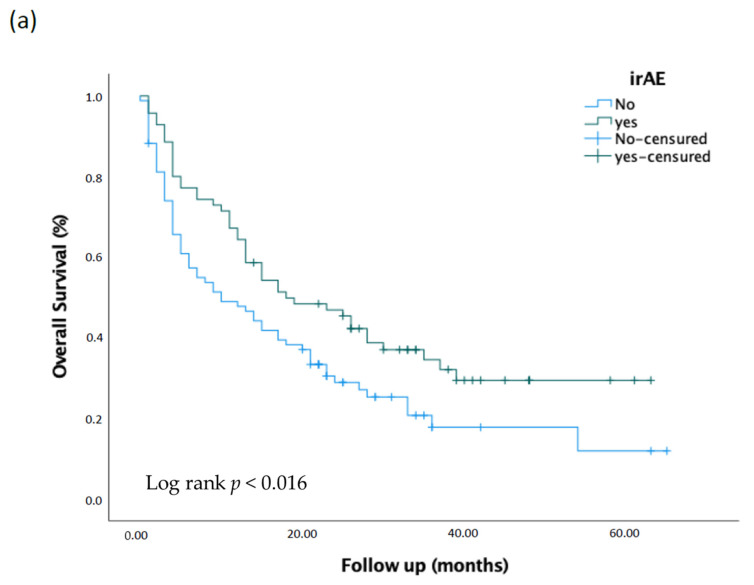

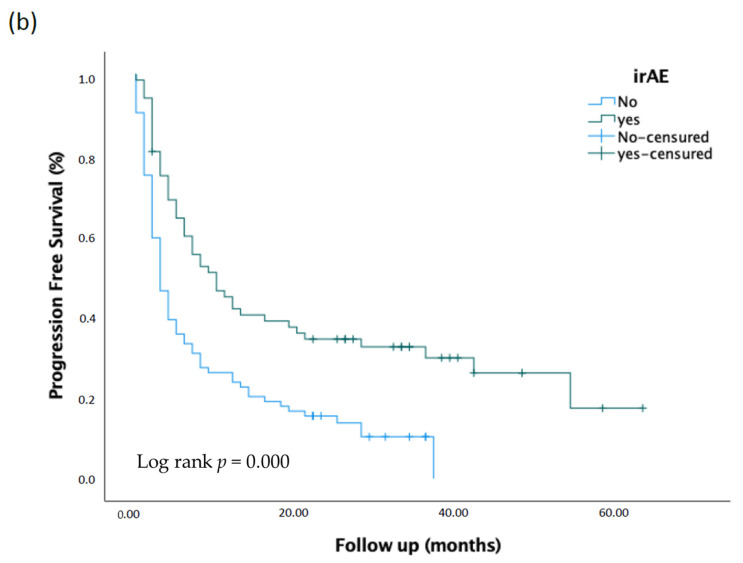
Kaplan–Meier analysis of overall survival (**a**) and progression free survival (**b**) comparing patients irAE and non-irAE.

The Kaplan–Meier estimates for overall survival and progression-free survival for patients with irAE were compared to those without irAE, as shown in Figure 1. The results were significant when compared using the log-rank test. The median overall survival for patients with irAE was 18 months (95% CI, 8.67–27.32) compared to 10 months (95% CI, 3.48–16.52) for patients without irAE, with a *p*-value of <0.016. Similarly, the median progression-free survival was 10 months (95% CI, 5.44–14.56) for patients with irAE and three months (95% CI, 1.94–4.05) for patients without irAE, with a *p*-value of 0.000. 

In univariate analysis, the benefit of irAE persisted. The risk of death in patients with irAE was 33% lower compared to patients without such events (HR: 0.67; 95% CI, 0.46–0.99; *p* = 0.043). A Cox proportional hazards analysis was conducted to identify factors associated with increased mortality, as shown in Table 3. 

In multivariate analysis, irAE experience and ECOG were included as they were related to the outcome with statistical significance (*p* < 0.05) in univariate analysis. When adjusted for ECOG, irAE experience persisted as an independent prognostic factor associated with better overall survival (HR: 0.65; 95% CI, 0.44 to 0.96; *p* = 0.03).

## 4. Discussion

Despite the remarkable advantages of ICI witnessed over the last years, significant morbidity due to irAE can be a limiting factor for its widespread use and patients’ quality of life. Immune toxicity is unpredictable, diverse, and on several occasions disabling or life-threatening [16]. Overall, in this study, the treatment with ICI was well tolerated. In our sample, 45.2% of patients had an irAE of any grade, with fatal events in less than 1%, which is consistent with the published data [17,18,19,20,21]. The only fatal event was secondary to pneumonitis, which is in concordance with the results presented in a recent network meta-analysis where pneumonitis was also the most common cause of irAE grade 5 [22]. 

The most frequent adverse events by organ systems are in line with other real-world evidence. Yet, the frequency of neurological events could be considered higher than what was reported before [23,24]. This can be justified by heterogeneity and lack of standardization in irAE documentation/registration. While there is a consensus between the type of irAE and ICI class, as colitis and hypophysitis are more common with anti-CTLA4 and pneumonitis and thyroiditis with anti-PD-(L)1 therapy, this analysis was not performed in our study due to the low percentage of patients treated with anti-CTLA4 [25].

It is uncertain whether ICI toxicities are distinct from standard autoimmune diseases or if the manifestation of irAE is associated with treatment efficacy. However, the potential association between tumor response to ICI and increased risk of developing irAE has been hypothesized since it is believed that irAEs could represent to some level the intensification of the immune response, including anti-tumor immune activity. While an early analysis of patients with melanoma treated with ipilimumab did not show a relationship between PFS and irAE, the following studies have associated a higher response rate with irAE incidence [26,27]. Data regarding outcomes within a single disease entity and irAE are more limited but have been shown in melanoma, NSCLC, and more recently in urothelial and hepatocellular carcinomas [28,29,30,31].

In our study, we observed a strong correlation between the development of irAE and patients’ response rates as well as PFS and OS. These results add to the robust growing evidence suggesting better oncological outcomes in patients who develop irAE. Although most patients included had a diagnosis of NSCLC, there was no correlation between tumor type and the outcomes. 

Therefore, a positive relation between irAE incidence and better clinical outcomes in cancer patients is almost certain, although not completely validated. There are some questions to be further explored, such as the role of ethnicity. Ethnical discrepancies in the overall survival of cancer patients have been commonly observed [32]. Furthermore, it is known that genetic polymorphisms of PD-1 and CTLA-4 are associated with various autoimmune diseases, such as thyroiditis, diabetes mellitus, and rheumatoid arthritis [33]. As comprehensive registration trials might fail to identify racial side effect profile disparities, real-world data could help to overcome this issue. To the best of our knowledge, this is the first study in Portuguese patients and, as enlightened above, the ICI toxicity profile is in line with other populations. However, since it is believed that the Portuguese population has an important incidence of autoimmune diseases, a superior frequency and/or higher grade of irAE could be expected, as reported in a Finnish study [34,35]. Hence, the pathophysiology and genetic background underlying irAE need additional investigation. Recent studies have identified certain genetic variants that may be associated with increased risk for irAEs, as well as variants that may predict a better response to immunotherapy. However, more research is needed to fully understand the role of germline factors in immunotherapy treatment and to develop personalized treatment strategies for patients [36].

This study had some limitations. First, it was retrospective, leading to information bias. Secondly, population heterogeneity was wide. Thus, the weighted average outcomes should be interpreted accordingly. Third, data were analyzed using a log-rank test and a standard Cox model, which introduces a bias owing to the different follow-up times and treatment exposures between patients who did and did not develop irAE. As such, a time-dependent analysis could be an alternative to minimize that bias in future analysis. Fourth, our study is skewed towards patients treated for advanced or metastatic NSCLC and melanoma, which could be explained by the earlier approval of ICI use in these settings. Lastly, a vast majority of the patients were treated predominantly with anti-PD1 drugs, with very few patients receiving anti-PD-L1 or anti-CTLA-4 agents. Thus, it would be difficult to generalize our findings to patients that have received ICI other than anti-PD-1.

## 5. Conclusions

The development of irAE predicted better outcomes including OS in Portuguese patients with advanced solid tumors treated with ICI. Further prospective studies are needed to explore and validate this prognostic value. The long-term impact of immune checkpoint blockade on quality of life, the detrimental effect of steroid administration on anticancer efficacy, and the biological underlying mechanisms of irAE also need more investigation.

## Figures and Tables

**Table 1:** Patients and tumor characteristics at baseline in the whole cohort and specified by development or not of irAE.

Variables	Whole Cohort n (%)	irAE n (%)	non-irAE n (%)
**Age (years** **)**			
Median (range)	64 (21–86)	64 (36–86)	65 (21–85)
**Sex**			
Male	118 (76.1)	52 (74.3)	66 (77.6)
Female	37 (23.9)	18 (25.7)	19 (22.4)
**Tumor type**			
Lung, non-small cell	76 (49.0)	42(60)	34 (40.0)
Melanoma	28 (18.1)	16 (22.8)	12 (14.2)
Renal	18 (11.6)	7(10.0)	11 (12.9)
Head and neck	17 (11.0)	2 (2.9)	15 (17.6)
Bladder	11 (7.1)	3 (4.3)	8 (9.4)
Others	5 (3.2)	-	5 (6.0)
**ECOG PS**			
0	38 (24.5)	17 (24.3)	21 (24.7)
1	101 (65.2)	46 (65.7)	55 (64.7)
2	16 (10.3)	7 (10.0)	9 (10.6)
**Treatment Line**			
First	50 (32.3)	28 (40.0)	22 (25.9)
Second	91 (58.7)	37 (52.9)	54 (63.5)
Third	11 (7.1)	5 (7.1)	6 (7.1)
Fifth or later	3 (1.9)	-	3 (3.6)
**Type of Immune Checkpoint inhibitor**			
Anti-PD-1/PD-L1	146 (94.2)	66 (94.3)	80 (94.1)
Anti-CTLA-4	9 (5.8)	4 (5.7)	5 (5.9)

ECOG PS-Eastern Cooperative Oncology Group Performance Status; PD-1-programmed cell death 1; PD-L1-programmed cell death-ligand 1; CTLA-4-cytotoxic T lymphocyte-associated protein 4.

**Table 2:** Immune-related adverse events in the whole cohort.

Variables	n (%)
**Treatment-related irAEs**	
yes	70 (45.2)
no	85 (54.8)
**Grade of irAE**	
<3	91 (92.9)
≥3	9 (8.1)
**Frequency of irAEs**	
1	33(47.1)
2	26 (37.1)
3	11(15.71)
**Type of irAE**	
Dermatologic	34 (35.4)
Pruritus	18(18.7)
Rash	14(14.7)
Vitiligo	1(1.0)
Bullous pemphigoid	1(1.0)
Neurologic/Musculoskeletal	13 (13.5)
Myalgias	12 (12.5)
Immune-mediated necrotizing myopathy	1(1.0)
Endocrin	14(14.7)
Hypothyroidism	11 (11.5)
Hypertiroidism	3 (3.2)
Rheumatologic	17 (17.7)
Artralgias	16 (16.7)
Vasculitis	1(1.0)
Pulmonary	8(8.3)
Pneumonitis	8 (8.3)
Gastrointestinal and Hepatic and biliary	9 (9.4)
Diarrhea	2 (2.1)
Colitis	2 (2.1)
Hepatitis	3 (3.2)
Colangitis	1 (1.0)
Colestases	1 (1.0)
Renal	1 (1.0)
Nephritis	1 (1.0)
**Treatment of irAE**	
Supportive care	66 (94.3)
Oral Corticosteroid	27 (38.6)
Intravenous Corticosteroid	5 (7.1)
Other Immunosuppressor (Methotrexate)	1(1.4)

**Table 3:** Univariate and Multivariate cox proportional hazards regression analysis of the risk of death.

Predictable Variables	HR Crude (CI 95%)	*p* Value	HR Adjusted (CI 95%)	*p* Value
irAE Yes vs. No	0.67 (0.46–0.99)	0.043	0.65 (0.44–0.96)	0.03
Sex Male vs. female	0.73 (0.48–1.12)	0.152		
ECOG PS 1 vs. 0	1.84 (1.12–3.03)	0.017	1.81 (1.10–2.88)	0.020
2 vs. 0	3.50 (1.72–7.11)	0.001	3.73 (1.83–7.62)	0.000
Age <65 vs. ≥65	0.95 (0.65–1.30)	0.771		
Treatment Line 1 vs. ≥2	1.29 (0.85–1.98)	0.230		
Type of tumor NSCLC vs. other	0.75 (0.52–1.11)	0.151		
Grade of toxicity <3 vs. ≥3	0.46 (0.19–1.14)	0.094		

ECOG PS-Eastern Cooperative Oncology Group Performance Status; NSCLC-Non-Small Cell Lung Cancer.

## Data Availability

The datasets generated and/or analyzed during the current study are not publicly available. However, they are available from the corresponding author upon reasonable request.
